# Probing protein interactions in living mammalian cells on a microtubule bench

**DOI:** 10.1038/srep17304

**Published:** 2015-11-27

**Authors:** Mirela Boca, Dmitry A. Kretov, Bénédicte Desforges, Alix Mephon-Gaspard, Patrick A. Curmi, David Pastré

**Affiliations:** 1 Laboratoire Structure-Activité des Biomolécules Normales et Pathologiques, INSERM U1204 and Université Evry-Val d’Essonne, Evry, 91025 France; 2 Institute of Protein Research, Russian Academy of Sciences, Pushchino, Moscow Region, 142290 Russia

## Abstract

Microtubules are μm-long cylinders of about 25 nm in diameter which are present in the cytoplasm of eukaryotic cells. Here, we have developed a new method which uses these cylindrical structures as platforms to detect protein interactions in cells. The principle is simple: a protein of interest used as bait is brought to microtubules by fusing it to Tau, a microtubule-associated protein. The presence of a protein prey on microtubules then reveals an interaction between bait and prey. This method requires only a conventional optical microscope and straightforward fluorescence image analysis for detection and quantification of protein interactions. To test the reliability of this detection scheme, we used it to probe the interactions among three mRNA-binding proteins in both fixed and living cells and compared the results to those obtained by pull-down assays. We also tested whether the molecular interactions of Cx43, a membrane protein, can be investigated with this system. Altogether, the results indicate that microtubules can be used as platforms to detect protein interactions in mammalian cells, which should provide a basis for investigating pathogenic protein interactions involved in human diseases.

Deciphering the complex interaction network of proteins in cells remains a challenging issue to understand the cell metabolism and function. To probe whether a protein of interest interacts with putative partners, high-throughput methods like yeast two-hybrid system^1^,^2^ or combination of affinity purification with mass spectrometry analysis^2^,^3^ are commonly used. Although these methods are essential to explore protein interactions at large scale, it is necessary to control the relevance of the proposed interactions in a context closer to native conditions, such as in living mammalian cells.

Different methods are then currently used to control the results of large-scale screening. For example, the colocalization between two proteins detected by fluorescence microscopy may indicate a putative interaction in mammalian cells. However, the use of colocalization assays is restricted to proteins confined to specific compartments like vesicles^4^. Other techniques like proximity ligation^5^, split-GFP^6^ and fluorescent resonance energy transfer (FRET,^7^) have also been developed to probe protein interactions in cells. These approaches present both strengths and limitations. Proximity ligation assays can reveal direct interactions between endogenous proteins but only in fixed cells. In addition, a rolling circle amplification procedure is required for detection and can bias the results. FRET uses short-range energy transfer to detect interactions between specifically modified proteins expressed in cells. A dedicated equipment is however required for analyzing the FRET signal. Furthermore, the FRET signal strongly depends on the distance and orientation between the donor and acceptor molecules, which has to be taken into account. In split-GFP experiments, the detection scheme is simple as the association between complementary GFP fragments attached to both bait and prey proteins leads to fluorescence but their intrinsic affinity also induces an inherent background.

Here, we present a new method which uses the microtubule network as an intracellular platform to detect protein interactions in living cells. The principle is as follows: a bait protein is brought to microtubules so that it can still interact and attract its putative preys onto microtubules. The presence of a prey on microtubules then reveals an effective interaction. This method can work with various prey molecules including nucleic acids and proteins and can detect both direct and indirect interactions. Microtubules are ideal platforms to probe protein interactions at the level of single cell for two reasons. (i) The surface area offered by microtubules is potentially enormous which should prevent saturation by bait proteins in most cases. Indeed, if we consider the number of microtubules per cell^8^, simple math indicates that the surface area of microtubules is larger than about 70 μm^2^ in typical mammalian cells like HeLa cells. If the bait protein requires an interacting surface area of 10 nm^2^, virtually, more than 7 × 10^6^ bait copies per cell can be anchored to microtubules. (ii) The microtubule network has a characteristic filamentous structure, which is a major advantage. An interaction between bait and prey indeed leads to the appearance of this characteristic structure in the fluorescence image of the prey. Protein interaction on a microtubule bench can then be easily distinguished in the cell cytoplasm and should allow a highly sensitive detection scheme by using numerical tools.

To explore the validity of this method, we first considered whether Tau, a microtubule-associated protein, can help to bring a bait protein onto microtubules, which is a prerequisite for the present detection scheme. We then examined if interactions among mRNA-binding proteins can be detected by using microtubules as platforms. We also developed a protocol to analyze the interactions between bait and prey from fluorescence images of cells. We finally tested whether the present method can be used to probe the interaction of other proteins like membrane proteins.

## Results and Discussion

### Tau brings bait proteins onto microtubules and preserves the accessibility of baits to molecular partners

**Figure 1:**
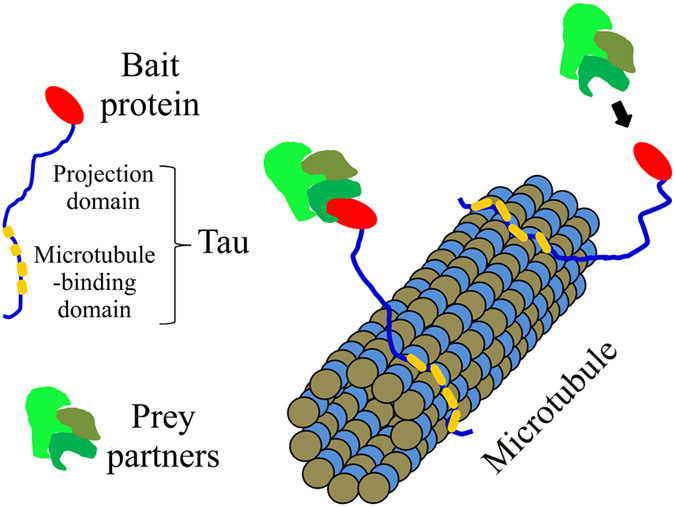
Schematic representation of the method used to detect protein interactions.

**Figure 2:**
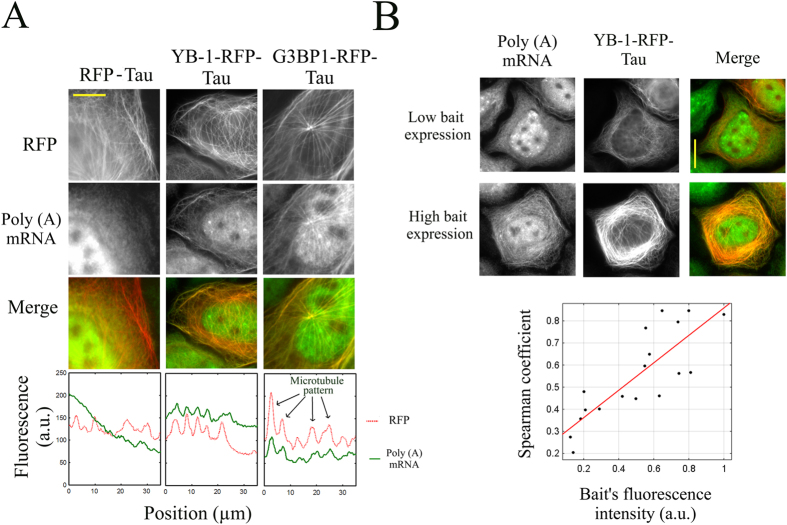
mRNA-binding proteins fused to Tau bring mRNAs onto microtubules in cells. (**A**) The micrographs show that two mRNA-binding proteins, YB-1 and G3BP1, when fused to Tau, brought mRNA onto microtubules. A fluorescent Poly(T) probe was used to detect Poly(A) mRNA. Tau-RFP is used as a control. Scale bar: 15 μm. In the presence of mRNA-binding proteins fused to Tau, representative line profiles reveal the binding of mRNA to microtubules. (**B**) Upper panel: fluorescence images of cells expressing YB-1-RFP-Tau at different levels reveal that the relocation of mRNA on microtubules is clearer at higher than lower bait expression level. Poly(A) mRNA was detected by using a fluorescent Poly(T) probe. Scale bar: 15 μm. Lower panel: the spearman coefficient, which reflects the degree of colocalization between mRNA and YB-1-RFP-Tau, used as bait, increases with the bait’s fluorescence intensity.

Various strategies can be considered to bring a protein onto microtubules and to use it as bait. Fusing the bait protein directly to tubulin, the building block of microtubules, may not be relevant since the concentration of free tubulin exceeds 10 μM in mammalian cells. Only a small fraction of bait proteins would thus be brought to microtubules^9^. Alternatively, microtubule-associated proteins (MAPs) like Tau can be used for this purpose (Fig. 1). Tau has a higher affinity for microtubules than for free tubulin^10^. Moreover, the bait protein can be fused to the unstructured N-terminal domain of Tau (11) which should keep the bait protein away from the microtubule surface. Increasing the spacing between the bait protein and the microtubule surface is indeed critical to preserve putative bait:prey interaction (Supplementary Figure S1A). We tested the efficiency of Tau fusion with two established mRNA-binding proteins, YB-1^11^ and G3BP1^12^, used as baits. Importantly, these two proteins have a diffuse distribution throughout the cytoplasm and were not found enriched on microtubules in HeLa cells (Supplementary Figure S1C). After their fusion to the N-terminal end of Tau with an additional fluorescent label, the two constructs, YB-1-RFP-Tau and G3BP1-RFP-Tau, were clearly brought to microtubules (Fig. 2A). To explore whether YB-1-RFP-Tau and G3BP1-RFP-Tau can still interact with their physiological substrate, mRNA, we probed the presence of poly(A) mRNAs on microtubules via *in situ* hybridization (Fig. 2A). The results indicate that the two bait proteins are able to bring large molecules such as mRNA on microtubules. As YB-1 interacts mostly with mRNA in cells^13^, we also explored whether mRNA preferential colocalizes with YB-1-RFP-Tau by comparison with ribosomal RNAs (Supplementary Figure S2A). To that end, we used the spearman coefficient^14^,^15^ , which reflects the colocalization level between bait and prey. It is closely-related to the Pearson coefficient^16^ but includes nonlinear relationship. As the fluorescence intensity on microtubules does not necessarily increase linearly with the number of fluorescent bait or prey, the spearman coefficient is therefore more adapted to the present analysis than the Pearson coefficient. The value of the spearman coefficient should normally range from 0 to 1, which reflects no apparent or perfect colocalization, respectively. A negative value indicates that the prey protein may be excluded from the microtubule vicinity. The measurements of the spearman coefficient reveal that YB-1-RFP-Tau significantly colocalizes with poly(A) mRNA but to a lesser extent with ribosomal RNAs (Supplementary Figure S2B). As expected, YB-1-RFP-Tau thus preferentially interacts with mRNA rather than with ribosomal RNAs on microtubules.

### The microtubule surface is not saturated by bait proteins even when expressed at high levels

A major advantage of using microtubules as platforms lies on the large surface area that they offer, which should theoretically preclude saturation by the bait protein. If saturation takes place, any bait protein in excess could capture a putative prey in the bulk cytoplasm. The chances of detecting bait:prey interactions on microtubules would therefore be reduced and in turn the sensitivity of the detection scheme would be impaired. However the surface area truly available on microtubules has not been reported so far. Microtubules interact with many other proteins like MAPs and molecular motors which can compete with the bait proteins for the binding to microtubules. To estimate the space available for bait proteins on microtubules, we measured the spearman coefficient reflecting the colocalization score between mRNA and YB-1-RFP-Tau for different expression levels of YB-1-RFP-Tau. If saturation arises above a critical expression level of YB-1-RFP-Tau, the correlation coefficient should no longer increase. However the spearman coefficient rather increases steadily with the expression level of YB-1-RFP-Tau which indicates that more mRNAs were brought to microtubules. The surface of microtubules was thus not saturated under the conditions tested (Fig. 2B).

### Microtubules can be used as platforms for detecting the interactions among mRNA-binding proteins

**Figure 3:**
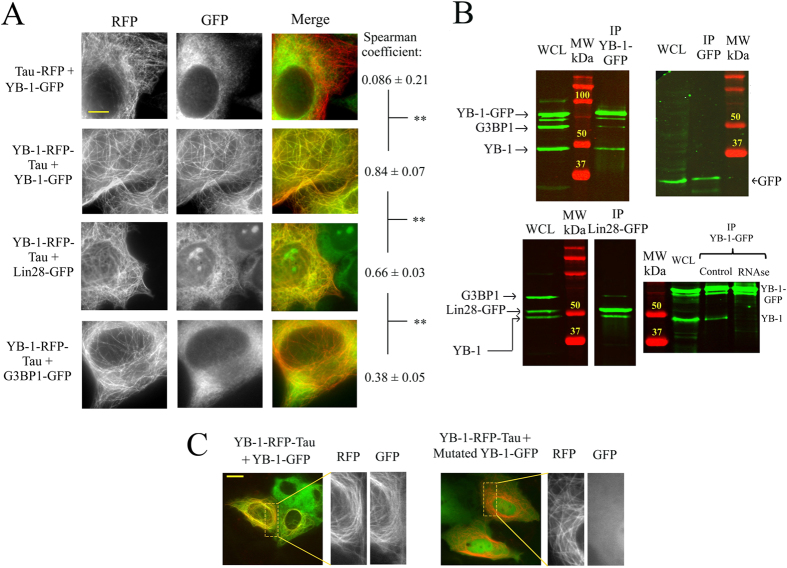
Detection of protein colocalization among mRNA-binding proteins and comparison to results obtained via conventional pull downs. (**A**) The micrographs of fixed cells show the level of colocalization of indicated baits and preys, RFP and GFP-labeled respectively. Tau-RFP is used as a control. Results are mean ± SD (n = 20 cells). **P < 0.01, two-tailed t test. Scale bar: 15 μm. See Videos 1 and 2 for living cells. (**B**) Analysis of the interactions between YB-1, G3BP-1 and Lin28 via pull down assays. Upper, left panel: western blots showing the amounts of endogenous YB-1, G3BP1 and YB-1-GFP in pull-down assays of whole cell lysate (WCL) of HeLa cells expressing YB-1-GFP. Anti-GFP antibody was used to bring YB-1-GFP to the beads. Upper, right panel: as a control, neither YB-1 nor G3BP1 were detected in pull-down assays using whole cell lysate of GFP-transfected cells. Endogenous YB-1 and G3BP1 were detected using anti-YB-1 and anti-G3BP1 antibodies. HeLa cells do not express endogenous Lin28. Lower, left panel: pull down assays in HeLa cells expressing Lin28-GFP. Lower right panel: western blots showing the amount of endogenous YB-1 and YB-1-GFP in pull-down assays of WCL of HeLa cells expressing YB-1-GFP with or without RNAse treatment. RNAse disrupts the interaction between YB-1-GFP and endogenous YB-1, in line with a colocalization resulting from a cooperative binding to mRNA^18^. (**C**) Videomicroscopy images of living cells. Two point mutations in the cold-shock domain disrupt YB-1-GFP colocalization with YB-1-RFP-Tau on microtubules. Scale bar: 15 μm.

To further document the method, we then probed in fixed and living cells the interactions of YB-1-RFP-Tau, used as bait, with protein partners belonging to cytoplasmic mRNA-binding proteins (Fig. 3A, videos 1 and 2). In this case, the detected interaction may result from a direct interaction between mRNA-binding proteins or from a significant overlap in their mRNA targets. Considering these parameters, we chose three different prey proteins: YB-1, G3BP1 and Lin28.

i) YB-1 is known to interact with itself^17^ and to bind to mRNA cooperatively^18^. As many copies of this protein should be interacting with the same mRNA due to its cooperative binding, we thus expect a strong colocalization between YB-1-RFP-Tau and YB-1-GFP on microtubules.

ii) G3BP1 has no known interaction with YB-1. In addition, none of the top 25 mRNA targets of G3BP1^19^ were found among the top 100 mRNA targets of YB-1^20^. G3BP1 may thus poorly colocalize with YB-1-RFP-Tau on microtubules.

ii) Lin28 (Lin28a) is a regulator of development timing. Lin28, like YB-1, binds to RNA cooperatively via its cold-shock domain^21^,^22^. In addition to these similarities with YB-1, about 25% of the top 100 mRNA targets of YB-1^20^ display at least one binding site for Lin28^23^. Lin28 may thus colocalize with YB-1-RFP-Tau on microtubules.

The results indicate that, as expected, YB-1-GFP is clearly brought to microtubules in cells expressing YB-1-RFP-Tau (Fig. 3A, Video 1). In contrast, G3BP1-GFP scarcely colocalizes with YB-1-RFP-Tau under the same conditions (Fig. 3A, Video 2). Lin28-GFP is also observed on microtubules of cells expressing YB-1-RFP-Tau but to a lesser extent than YB-1-GFP. The measured colocalizations are therefore specific to the prey proteins. Interestingly, while G3BP1-RFP-Tau also brings mRNA to microtubules (Fig. 2A), it fails to do the same with YB-1-GFP, Lin28-GFP and G3BP1-GFP (Supplementary Figure S4). The detected colocalization is thus not simply governed by the presence of mRNAs on microtubules. In order to compare these results with those obtained with a more conventional method, we performed pull-down assays. The results show that YB-1 indeed co-precipitates with itself and with Lin28 but poorly with G3BP1. In addition, we found that YB-1:YB-1 co-precipitation is RNA-dependent (Fig. 3B), in agreement with YB-1:YB-1 interactions resulting from a cooperative binding to RNA.

If we consider that the strong colocalization between YB-1-GFP and YB-1-RFP-Tau is due to the cooperative binding of YB-1 to mRNA^18^, the same results should be observed for Lin28 which was also reported to bind to RNA cooperatively *in vitro*^21^. We thus tested this hypothesis by using Lin28-RFP-Tau as bait and Lin28-GFP as prey. The results clearly reveal the presence of Lin28-GFP on microtubules in Lin28-RFP-Tau expressing cells (Supplementary Figure S4). In contrast, G3BP1, which does not bind to mRNA cooperatively^18^, poorly colocalizes with G3BP1-RFP-Tau on microtubules (Supplementary Figure S4).

### The consequences of point mutations on protein interactions can be probed on a microtubule bench

One possible application of this method is to detect the consequences of point mutations on protein interactions, which could pave the way for future structural biology investigations in cells. To that end, we probed the consequence of two point mutations in the cold-shock domain of YB-1^24^. These two mutations might play a critical role in YB-1:YB-1 interactions as shown by pull-down assays (Figure S3). We thus examined the interactions between YB-1-RFP-Tau, used as bait protein, and YB-1-GFP with or without the two mutations in the cold-shock domain^24^, (Fig. 3C). In contrast with wild type YB-1, the results show that mutated YB-1-GFP fails to colocalize with YB-1-RFP-Tau on microtubules.

### A protocol to analyze protein interactions on a microtubule bench

**Figure 4:**
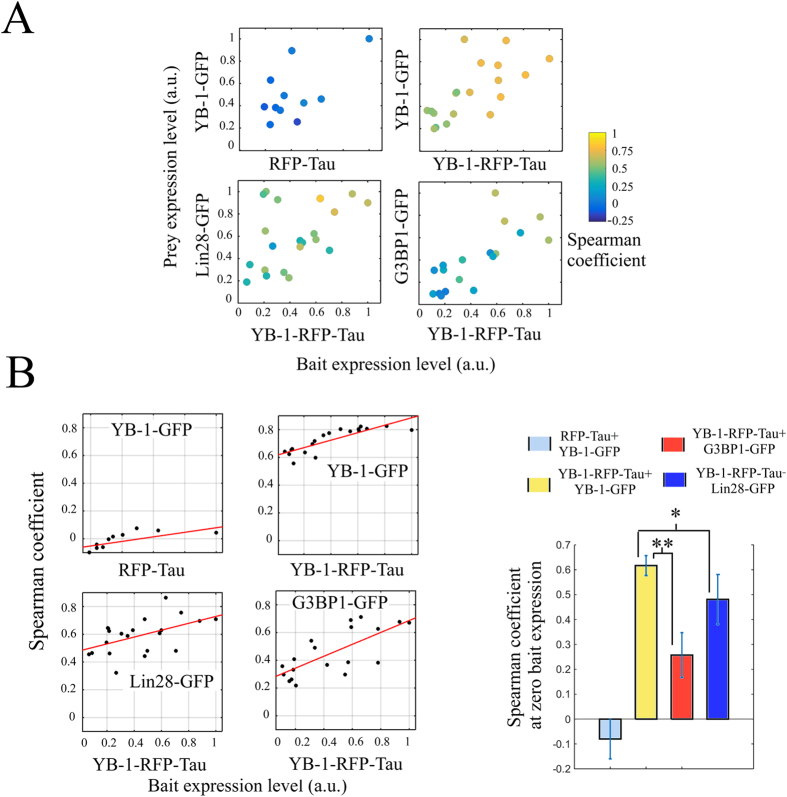
Statistical analysis of the colocalization events detected on microtubules. (**A**) 2D plot representing the spearman coefficient versus bait and prey expression levels. Each data point represents the mean value obtained on three different areas in the same cell. The color scale bar indicates the value of the spearman coefficient for each data point in the 2D plot. (**B**) 2D plot representing the spearman coefficient versus bait expression level. The value of the spearman coefficient when the bait expression level is virtually zero was then extrapolated using linear curve fitting. The bar plot represents the spearman coefficients extrapolated at the zero bait expression level. Results are means ± SD and were obtained from least square interpolation of the experimental data points (n ≥ 19 cells). **P < 0.01, *P < 0.05, two-tailed t test.

In order to design a protocol for measuring bait:prey interactions on microtubules, we considered that high expression levels of the bait protein may lead to false positive colocalizations. Indeed, a significant fraction of mRNA is then brought to microtubules, including non-specific mRNA targets of the bait protein. As a result, other RNA-binding proteins which do not colocalize with the bait protein under physiological conditions may be brought to microtubules artificially. To quantify such bias and discard false positive colocalizations, the spearman coefficient was measured in cells expressing both bait and prey proteins at varying levels (Fig. 4A,B). For both YB-1-RFP-Tau, Lin28-RFP-Tau and G3BP-RFP-Tau, the results indicate that the spearman coefficient increases gradually with the bait expression levels. At the highest expression levels of YB-1-RFP-Tau, the differences in the measured spearman coefficients between the three prey proteins are less marked than at low bait expression levels (Fig. 4B). This was expected on the basis of the massive binding of mRNA on microtubules after expressing YB-1-RFP-Tau at high levels (Fig. 2B). In line with this, when RFP-Tau alone was used as a control, which thus does not bring mRNA to microtubules (Fig. 4A), colocalization between YB-1-GFP and Tau-RFP remains insignificant whatever the expression level of RFP-Tau (Fig. 4B).

To avoid the bias induced by expressing the bait protein at high levels, we decided to estimate the spearman coefficient when the expression level of the bait protein is virtually zero, which corresponds to conditions found in unperturbed cells. The value of the spearman coefficients in the virtual absence of bait protein were then extrapolated from the plots of the spearman coefficient versus bait expression level (Fig. 4B). This analysis shows that, in unperturbed cells, YB-1-RFP-Tau significantly interacts with YB-1-GFP, moderately with Lin28-GFP and poorly with G3BP1-GFP.

### Protein interactions can be detected during microtubule regrowth after nocodazole washout

**Figure 5:**
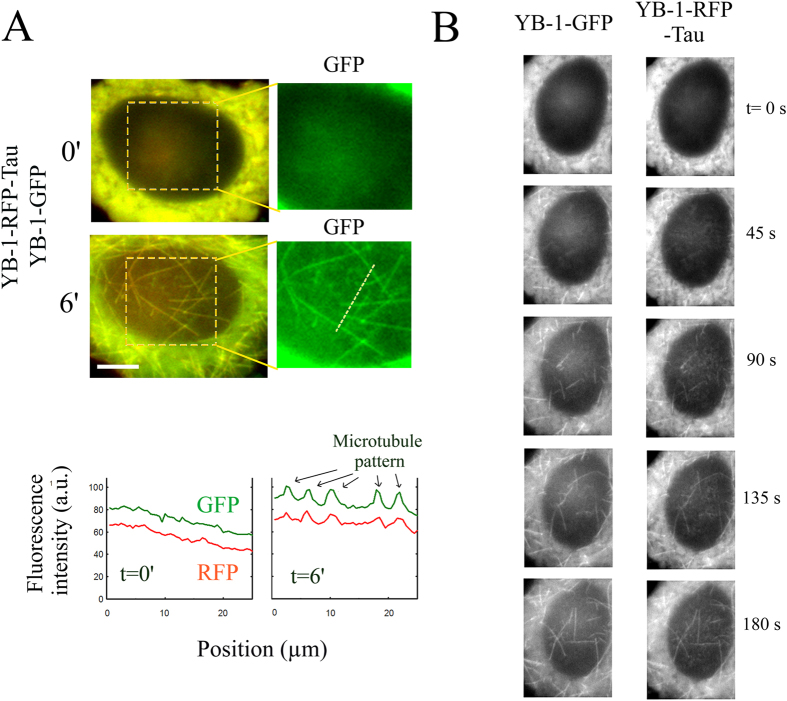
Detection of protein interaction in growing microtubules. (**A**) Upper panel: Re-growth of microtubules after nocodazole wash-out leads to the appearance of microtubule structures in both bait and prey time-lapse images. Scale bar: 15 μm. Lower panel: the line profile represents the fluorescence intensity along the dashed line (see upper panel) at two different times. Arrows indicate the position of five microtubules in the line profile at t = 6′. (**B**) Time-lapse imaging of the reforming microtubules after nocodazole wash-out. We noticed the simultaneous presence of YB-1-GFP and YB-1-RFP-Tau on dynamical microtubules. See also video 3.

So far the series of experiments show that the preys, whatever they are mRNA or mRNA-binding proteins, can be brought onto the microtubule surface when a bait protein is intentionally located on microtubules. However, even by using the N-terminal domain of Tau as spacer, the vicinity of the microtubule surface may hinder native interactions between bait and prey. To overcome this possible bias, we monitored microtubule disassembly/assembly by using reversible microtubule-destabilizing drugs like nocodazole or through cold treatment. Indeed, inducing microtubule disassembly/assembly opens the opportunity to let the bait-prey partner interact freely in the cytoplasm for a chosen time interval. The bait-prey couple can then be again attracted to nascent microtubules when conditions for microtubule assembly are again favorable. As a proof of concept, microtubules were first depolymerized in the presence of nocodazole and microtubule regrowth was then triggered after nocodazole washout. Using YB-1-RFP-Tau as bait and YB-1-GFP as prey, we then detected the presence of both bait and prey proteins on growing microtubules (Fig. 5A). Time-lapse images and videos (Fig. 5B and video 3) do not reveal any delay between the appearance of YB-1-RFP-Tau and that of YB-1-GFP on microtubules.

### Cx43 interacts on microtubules with a truncated form of Cx43, used as bait

**Figure 6:**
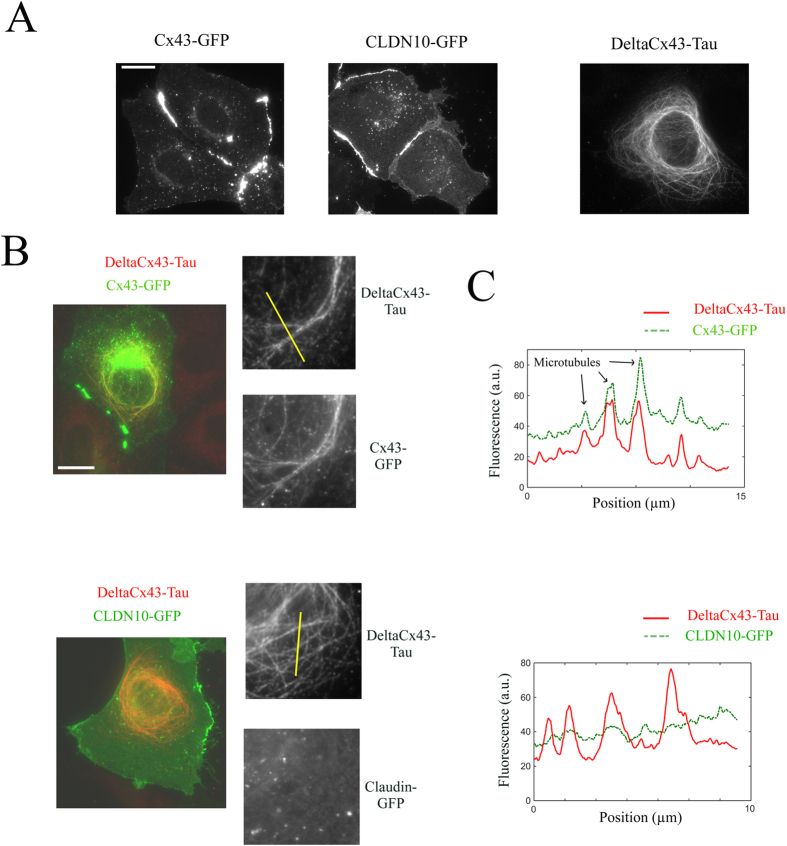
Interactions between membrane proteins on a microtubule bench. (**A**) Spatial distribution of Cx43-GFP, CLDN10-GFP (Claudin-10) and DeltaCx43-Tau (amino acids 147–382 of Cx43) in NRK cells. Both Cx43-GFP and CLDN10-GFP are located at the cell membrane interface. We also noticed that Cx43-GFP forms large gap junction plaques between cells. DeltaCx43-Tau was located on microtubules and can thus be used as bait protein. Anti-Tau was used to detect DeltaCx43-tau. Scale bar: 30 μm. (**B**) Fluorescence imaging of NRK cells expressing DeltaCx43-Tau, the truncated form of Cx43, and either Cx43-GFP or CLDN10-GFP. DeltaCx43-Tau, used as bait, is clearly brought to microtubules. Higher magnification images reveal the presence of Cx43-GFP on microtubules. In contrast, CLDN10-GFP does not colocalize with DeltaCx43-Tau on microtubules under similar conditions. NRK cells were stained with anti-Tau antibody to reveal the presence of DeltaCx43-Tau. Scale bar: 30 μm. (**C**) Representative line profiles reveal the spatial correlation between Cx43-GFP and DeltaCx43-Tau on microtubules. However, no spatial correlation was observed between CLDN10-GFP and DeltaCx43-Tau.

We also tested whether the domain of application of the present method can be extended to proteins other than RNA-binding proteins. Cx43, a membrane protein and a member of the connexin family, was chosen because it can oligomerize into hexamers at the cell membrane to form gap junctions. Here we used as bait protein a truncated form of Cx43, DeltaCx43, in which part of the N-terminal domain has been removed (amino acids 1-146). This truncated form contains two transmembrane helices but doesn’t form gap junctions^25^ and thus was easily brought to microtubules after fusing it to Tau (Fig. 6A). We then probed whether DeltaCx43-Tau, used as bait, interacts with the full length Cx43-GFP on microtubules. In epithelial NRK cells, Cx43-GFP was located at the cell membrane and formed gap junctions (Fig. 6A). Interestingly, when NRK cells expressed the bait protein, DeltaCx43-Tau, Cx43-GFP was also found on microtubules (Fig. 6B). The presence of CX43-GFP on microtubules should thus result from an interaction between DeltaCx43 and Cx43-GFP, probably through their transmembrane helices^26^. As a negative control, we used Claudin-10 (CLDN10), a member of the Claudin family^27^, which forms tight junction at the interface between cells. CLDN10 is not known to interact with Cx43. In line with this, CLDN10-GFP was found at the cell membrane and failed to colocalize with DeltaCx43-Tau on microtubules.

Altogether, the results show that the present method is particularly suitable to screen putative partners of proteins having a diffuse cytoplasmic distribution. Albeit it may also apply to proteins having a non-diffuse cytoplasmic distribution and to non-cytoplasmic proteins, careful attention should be paid to avoid false positives induced by changing the natural location of proteins. In addition, only cells expressing bait protein at low level should be taken into account to avoid considering nonspecific interactions as stressed by statistical approach (Fig. 4). Further developments may concern the combination of this method with fluorescence energy transfer FRET^28^ or split-GFP^6^ in order to determine whether the interactions taking place on the microtubule platform are direct. High throughput data can be possibly collected using this method but requires optimal lateral resolution with oil-immersed lenses to clearly distinguish microtubules in both fixed and living cells.

## Materials and Methods

### Cell culture

HeLa and NRK cells (Normal Rat Kidney cells) were obtained from the American Type Culture Collections (ATCC) and cultured at 37 °C in a humidified atmosphere with 5% CO_2_ in DMEM (Life Technologies) supplemented with 5% FBS (Life Technologies). Cells were grown on 12 mm round coverslips inside 24-well plates and were transfected with 2 μg of indicated DNA plasmids by using Lipofectamine 2000 (Invitrogen). For videomicroscopy, cells were cultured on glass bottom dishes (MatKek Corporation). The efficiency of transfection and the integrity of the encoded proteins were controlled by immunoblotting experiments (Supplementary Figure S1B).

### Molecular biology and plasmid preparation

The bait protein was fused to the longest isoform of the human Tau protein (Accession number: NP_005901.2) which has the longest N-terminal projection domain. The projection domain can possibly favor bait accessibility to preys. To explore this hypothesis, a truncated form of Tau was generated by removing the first 170 amino acids of the N-terminal tail of Tau. The Tau cDNA containing at its 5′ end the PacI, AscI and SphI restriction sites was amplified by PCR and inserted into the Gateway pCR8/GW/TOPO entry plasmid (Invitrogen™). The resulting plasmid will be mentioned hereafter as the “backbone entry plasmid”. Human YB-1, G3BP1, Lin28 (Lin28a) and DeltaCx43 cDNAs were amplified by PCR using primers containing the PacI and AscI restriction sites and then inserted into the pCR-Blunt II-TOPO plasmid (Invitrogen™). GFP or RFP cDNAs were amplified by PCR using primers containing the AscI and SphI restriction sites and then inserted into the pCR-Blunt II-TOPO plasmid (Invitrogen™) using standard protocols. The plasmids containing all the above mentioned cDNAs were propagated and purified by Thermo Scientific Mini Kit (reference K0503) and the inserted cDNAs were verified by sequencing. Human YB-1, G3BP1, Lin28 and DeltaCx43 cDNAs inserted into the pCR-Blunt II-TOPO plasmid were digested with PacI and AscI. GFP and RFP cDNAs inserted into pCRBlunt II-TOPO plasmid were digested with AscI and SphI. Ligation was performed in order to insert the above mentioned cDNA sequences into the backbone entry plasmid, previously digested with the same restriction enzymes (PacI, AscI and SphI).

The following entry plasmids were then generated:

RFP-Tau-pCR8/GW/TOPO, YB-1-RFP-Tau-pCR8/GW/TOPO, G3BP1-RFP-Tau-pCR8/GW/TOPO, Lin28-RFP-Tau-pCR8/GW/TOPO, DeltaCx43-Tau-pCR8/GW/TOPO.

The LR recombination reactions (Invitrogen™) were performed according to the manufacturer’s protocol in order to transfer the cDNAs of interest from the backbone entry plasmids into the Gateway® pEF-Dest51 plasmid (Invitrogen™) suitable for protein expression in eukaryotic cells. Finally, the following expression plasmids were also generated:

RFP-Tau-pEF-Dest51, YB-1-RFP-Tau-pEF-Dest51, Lin28-RFP-Tau-pEF-Dest51, G3BP1-RFP-Tau-pEF-Dest51. DeltaCx43-Tau-pEF-Dest51.

For the preparation of the GFP-constructs, human YB-1, G3BP1, Lin28 (Lin28a), Cx43 and CLDN10 (isoform B) cDNAs were amplified by PCR and inserted into the pEGFP-N1 plasmid (Clontech). The inserted cDNAs were verified by sequencing. The double Y72A/F74A YB-1 mutant was obtained via site-directed mutagenesis.

### Fixed cell preparation

Cells growing on glass coverslips were washed with PBS, fixed with ice-cold methanol for 30 min at −20 °C, washed with PBS and then further fixed with 4% paraformaldehyde (PFA) in PBS for 45 min at 37 °C. This double Methanol/PFA fixation was preferred as it best reveals microtubule structures and colocalization events (Supplementary Figure S5). After final washes with PBS, samples were prepared for fluorescence microscopy imaging.

### Videomicroscopy of living cells

Cells were transiently transfected with the indicated expression plasmids and then cultured for 24 h before real-time monitoring of microtubule dynamics. Fluorescence videomicroscopy was implemented on an inverted microscope (Axiovert 220; Carl Zeiss 5 MicroImaging, Inc). GFP and (or) RFP emission was detected with a 63×/1.4 NA objective. Time-lapse images were captured at indicated time intervals using a cooled CCD camera (Zeiss).

### Image analysis and Statistics

Cells were co-transfected and analyzed after 36 h when expressing both bait and prey tagged with two different fluorescent labels, RFP and GFP, respectively. Only cells with a healthy appearance were selected for the statistics. In the case of cells expressing G3BP1-GFP, cells displaying stress granules were discarded. This occurs at high expression level of G3BP1-GFP. We also paid attention to obtain optimal resolution conditions and to select cells in which microtubules were clearly distinguished using the bait’s fluorescence as the signal. For the same series of experiment, we used the same objective lens (100 × /1.4 NA or 65 × /1.4 NA for fixed and living cells respectively). At this stage, we analyzed whether, although using achromatic lenses, the red and green images were not shifted with respect to each other. Such shift, if any, can be corrected using the ImageJ’s Plug-In, “Align RGB planes” (Supplementary Figure S6). To quantify the colocalization level between a protein bait fused to Tau and putative protein preys, we adapted a method previously described^16^. Both images were then filtered using a FFT high pass filter to remove spatial frequencies which are not relevant to microtubule structures (larger structures than 2 μm). Images of the bait and the prey were then merged into a single green-red image. Then, the ImageJ’s plug-in, “PSC Colocalization”, was used to measure the spearman’s coefficient, in three different regions of interest (ROI) for the same cell where microtubules were clearly observed in the bait image (Supplementary Figure S6). The area of the ROI was fixed to avoid any bias due to the surface considered to measure the correlation coefficient. We controlled that all the experimental results presented in this article were reproducible by performing each experiment in triplicate. To extrapolate the value of the spearman coefficient at the zero expression level of the protein bait, we used the least square method and a linear curve fitting.

### Microtubule regrowth assay

Cells were placed on ice for 30 min to totally dissociate microtubules into tubulin heterodimers and then warmed-up at 37 °C in the presence of 300 nM nocodazole. Nocodazole was then washed out from the culture medium. After nocodazole removal, *de novo* microtubule elongation started from the centrosomal area.

### RNA hybridization *in situ*

*In situ* hybridization was performed to reveal Poly(A) mRNA and the 40S or 80S ribosomal subunits in HeLa cells as follows. Cells were fixed with cold methanol and then with PFA at 37 °C. Cells were incubated with 100% ice-cold methanol for 15 minutes at −20 °C, after that in ice-cold 70% ethanol for 10 minutes at −20 °C, and then 1 M Tris pH 8 for 5 minutes, before addition of a Cy2-conjugated oligonucleotides (Sigma) at 1 μg/μL in the hybridization buffer (0.005% BSA, 1 mg/mL yeast RNA, 10% dextran sulphate, 25% formamide in 2XSSC). 40 nucleotides Poly(T), 5′-AAGGATTTAA-AGTGGACTCATTCCAATTAC and 5′GGAT-TCTGACTTAGAGGCGTTCAGTCATAA probes, were used to detect mRNA and the 18S (40S subunit) and 28S (80S subunit) rRNA in cells respectively. Slides were then placed in a humidity chamber for 1 h at 37 °C with gentle shaking. Following hybridization, cells were washed twice with 4X SSC and once with 2XSSC.

### Pull down assays

The co-immunoprecipitation assays were performed using Dynabeads® Protein G Kit (Invitrogen, cat n° 10007D). HeLa cells were transiently transfected with YB-1-GFP, Lin28-GFP and GFP as a control. After 24h the cells were rinsed with ice-cold PBS, incubated on ice in cell lysis buffer (25mM Tris, 150mM NaCl, 1mM EDTA, complete Protease Inhibitor Cocktail and 1% Triton-X, pH 7.4) for 15 minutes, then scraped off the plates and centrifuged for 20 minutes at 15,000 × g, 4 °C. Aliquots of 25 μL of the whole cell lysate (WCL) of YB-1-GFP, Lin28-GFP and GFP were analyzed by Western blotting. 200 μL of cell lysate (1mg/ml proteins content), 2 μg mouse antiGFP primary antibody (ROCHE, 11814460001) and 50 μL Dynabeads® Protein G were mixed and incubated with gentle rocking overnight at 4 °C. The next day, the Dynabeads-Ab-Ag complexes were washed once with 200 μL lysis buffer, twice with 200 μL washing buffer (Citrate-Phosphate buffer, pH 5.0), then resuspended in 100 μL washing buffer and transferred to clean tubes in order to avoid co-elution of proteins bound to the tube wall. The Target Antigens (YB-1-GFP, Lin28-GFP and GFP) were eluted in 20 μL of elution buffer (0.1 M citrate, pH 2-3) and the pH of the eluates was adjusted by adding 1 M Tris, pH 7.5.

Endogenous YB-1 and G3BP1 were detected using anti-YB-1 and anti-G3BP1 (Sigma-Aldrich, G6046) antibodies. Anti-GFP (Thermo Scientific, PA1-980A) was used to detect YB-1-GFP and Lin28-GFP. Lin28 is not expressed endogenously in HeLa cells. The Precision Plus Protein Kaleidoscope standards (Bio-Rad, 161-0375) was used to determine the molecular weights of the following proteins: endogenous YB-1 and G3BP1, YB-1-GFP and Lin28-GFP.

## Additional Information

**How to cite this article**: Boca, M. *et al.* Probing protein interactions in living mammalian cells on a microtubule bench. *Sci. Rep.* **5**, 17304; doi: 10.1038/srep17304 (2015).

## Supplementary Material

Supplementary Information

Supplementary Video 1

Supplementary Video 2

Supplementary Video 3

Supplementary Figure S1

Supplementary Figure S2

Supplementary Figure S3

Supplementary Figure S4

Supplementary Figure S5

Supplementary Figure S6
