# Supplementary material for: Probing protein interactions in living mammalian cells on a microtubule bench
Source: Sci Rep. 2015 Nov 27;5:17304. doi: 10.1038/srep17304 (PMC4661529; doi:10.1038/srep17304)
Supplement: Supplementary Information [file srep17304-s1.pdf]

Probing protein interactions in living mammalian cells on a microtubule bench

Mirela Boca[1], Dmitry A Kretov[1,2], Bénédicte Desforges[1], Alix Mephon-Gaspard[1], Patrick A Curmi[1] and David Pastré [1,*]

LEGEND TO SUPPLEMENTARY FIGURES AND VIDEOS:

Supplementary Figure S1: The projection domain of Tau increases the occurrence of colocalization between bait and prey.

A) The projection domain of Tau increases the occurrence of colocalization between bait and prey. YB-1 was fused to full length or N-terminally truncated Tau and used as bait. The scatter plot shows the spearman coefficients for varying bait and prey expression levels. This coefficient represents the colocalization score for the bait and prey considered. The plot representing the spearman coefficient versus the bait fluorescence clearly indicates that the N-terminal truncation of Tau has a negative impact on the level of colocalization between YB-1-RFP-Tau and YB-1-GFP.

B) Western blot analysis of HeLa cells expressing G3BP1-RFP-TAU, YB-1-RFP-TAU, Lin28-RFP-TAU. The molecular weights of G3BP1, YB-1 and Lin28 are about 52, 36 and 23 KD respectively. The molecular weight of TAU and RFP are 46 and 26 KD. Anti-Tau was used to detect the constructs.

C) G3BP1-GFP and YB-1-GFP have both a diffuse and cytoplasmic distribution in HeLa cells.

Supplementary Figure S2: mRNA is preferentially brought to microtubules by YB-1-RFP-Tau.

In cells expressing YB-1-RFP-Tau, the presence of mRNA and the 28S and 18S ribosomal subunits was probed via in situ hybridization. The bar plot represents means ± SD obtained from experimental measurements of the spearman coefficient (n=30 cells). $^{**}P<0.01$; two-tailed t test. Colocalization between YB-1-RFP-Tau and RNA was more pronounced in the case of messenger than ribosomal RNA as shown in line profiles and in the bar plot. While performing these measurements, we considered that ribosomal RNAs are much more numerous than mRNAs and are thus proportionally less likely to be bound to the bait protein on microtubules. To prevent such bias, the measurement of the correlation includes a filtering step that removes the background signal and alleviates such putative bias in the measurements (see materials and methods).

Supplementary Figure S3: Investigations on the mechanism of YB-1 self-interaction.

Western blots showing the presence of endogenous YB-1 and YB-1-GFP, with or without mutations in the cold-shock domain (Y72A/F74A), after pull down assays. Whole cell lysates (WCL) of HeLa cells expressing either wild type or mutated YB-1-GFP were used. The two point mutations alter YB-1 self-interaction as observed in cells (Figure 3C).

Supplementary Figure S4: Colocalization assays in HeLa cells reveal that Lin28 colocalizes with itself, in contrast with G3BP1.

Micrographs show HeLa cells expressing different combinations of bait and prey proteins. YB-1-GFP is not detected on microtubules in cells expressing G3BP1-RFP-Tau. In addition, G3BP1-GFP is not attracted onto microtubules using G3BP1-RFP-Tau as bait. Interestingly, Lin28-RFP-Tau brings Lin28-GFP to microtubules.

Supplementary Figure S5: Methanol fixation followed by PFA treatment best reveals colocalization on microtubules in fixed HeLa cells.

A) Methanol fixation followed by para-formaldehyde treatment is the most efficient fixation procedure to measure colocalization on microtubules in fixed cells.

Cells expressing YB-1-RFP-Tau and YB-1-GFP were fixed using either:

- 4% paraformaldehyde (PFA) for 45’ at 37°C
- ice-cold methanol for 30’
- ice-cold methanol for 30’ followed by PBS wash and 4% PFA treatment for 45’ at 37°C

Images of living cells were used as a control.

B) Plots represent representative line profiles of fluorescence signals. Microtubules were not clearly observed in the bait and prey images using PFA fixation or methanol alone. In the case of Methanol alone, Tau may dissociate from microtubules after the fixation step while preparing the sample for fluorescence microscopy.

Supplementary figure S6:

A) Detailed protocol to quantify colocalization between bait and prey on microtubules. Images of bait and prey florescence, here YB-1-RFP-Tau and YB-1-GFP, obtained from fixed cells, were first spatially filtered by using Fast Fourier Transform (FFT). Low spatial frequencies, corresponding to feature larger than 2 µm, were discarded. Microtubule structures

then appeared clearly in the images of the bait protein and, provided that colocalization occurs, in the images of the prey protein. Images of the bait and the prey were merged and proceed using the ‘Pearson-Spearman Correlation Colocalization’ plug-in for ImageJ.

B) The measured correlation coefficient is very sensitive to the alignment of the bait and prey fluorescence images. When images are shifted, the ImageJ’s plug in, ‘Align RGB planes’, can be used to correct the shift. The spearman coefficient representative of the colocalization between YB-1-GFP and YB-1-RFP-Tau then increases.

Videos 1 and 2: Time-lapse videomicroscopy of cells co-expressing:

1) YB-1-RFP-Tau and YB-1-GFP

2) YB-1-RFP-Tau and G3BP1-GFP

The images were acquired at 20 s intervals for 300 s with a digital camera.

Video 3: Time-lapse videomicroscopy of cell expressing YB-1-RFP-Tau and YB-1-GFP. The images were acquired at 45 s intervals for 675 s after nocodazole was removed from the culture medium to allow microtubule polymerization.
