# Supplementary figures and images for: Probing protein interactions in living mammalian cells on a microtubule bench
Source: Sci Rep. 2015 Nov 27;5:17304. doi: 10.1038/srep17304 (PMC4661529; doi:10.1038/srep17304)

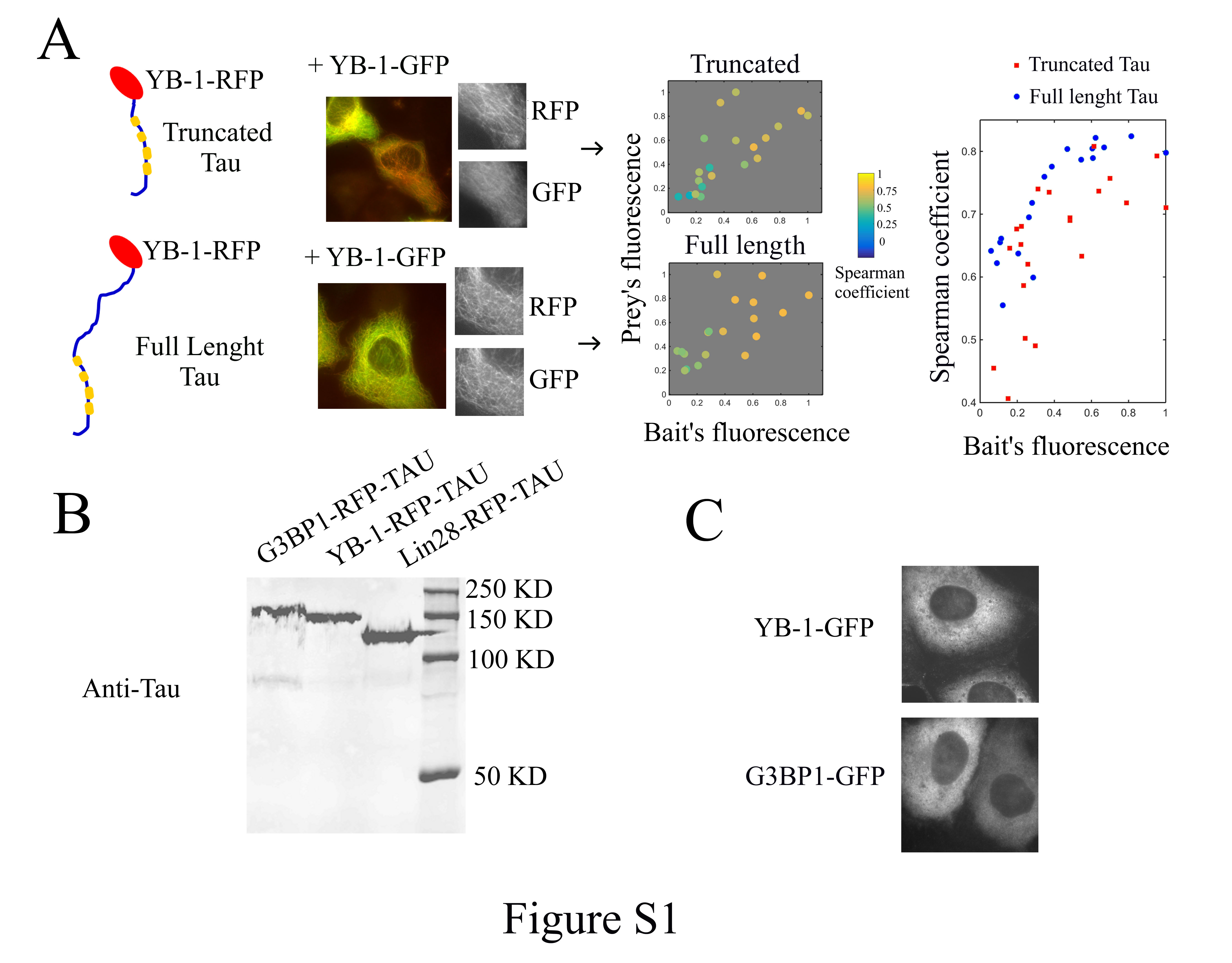

Supplement: Supplementary Figure S1 [file srep17304-s5.jpg]

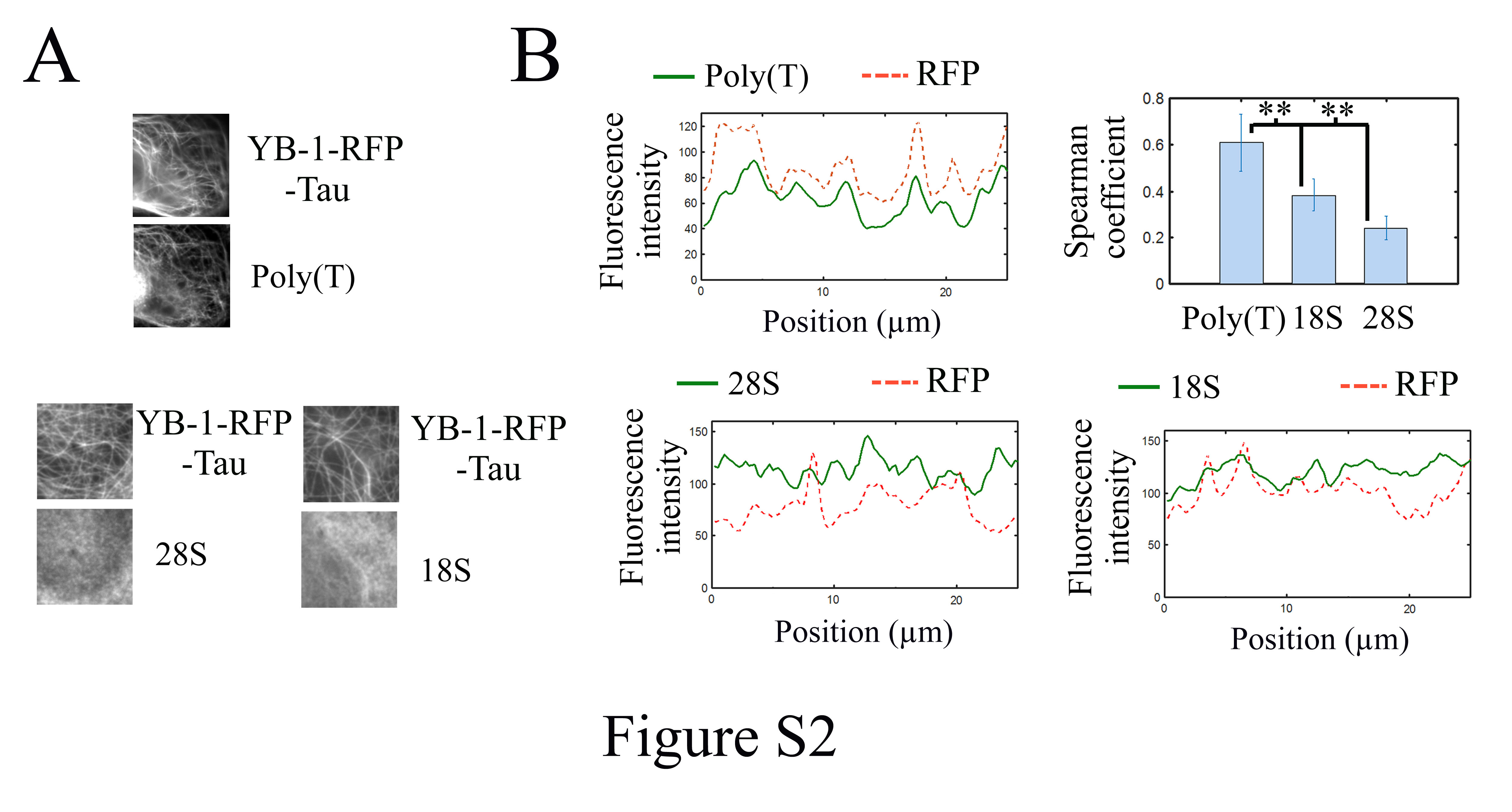

Supplement: Supplementary Figure S2 [file srep17304-s6.jpg]

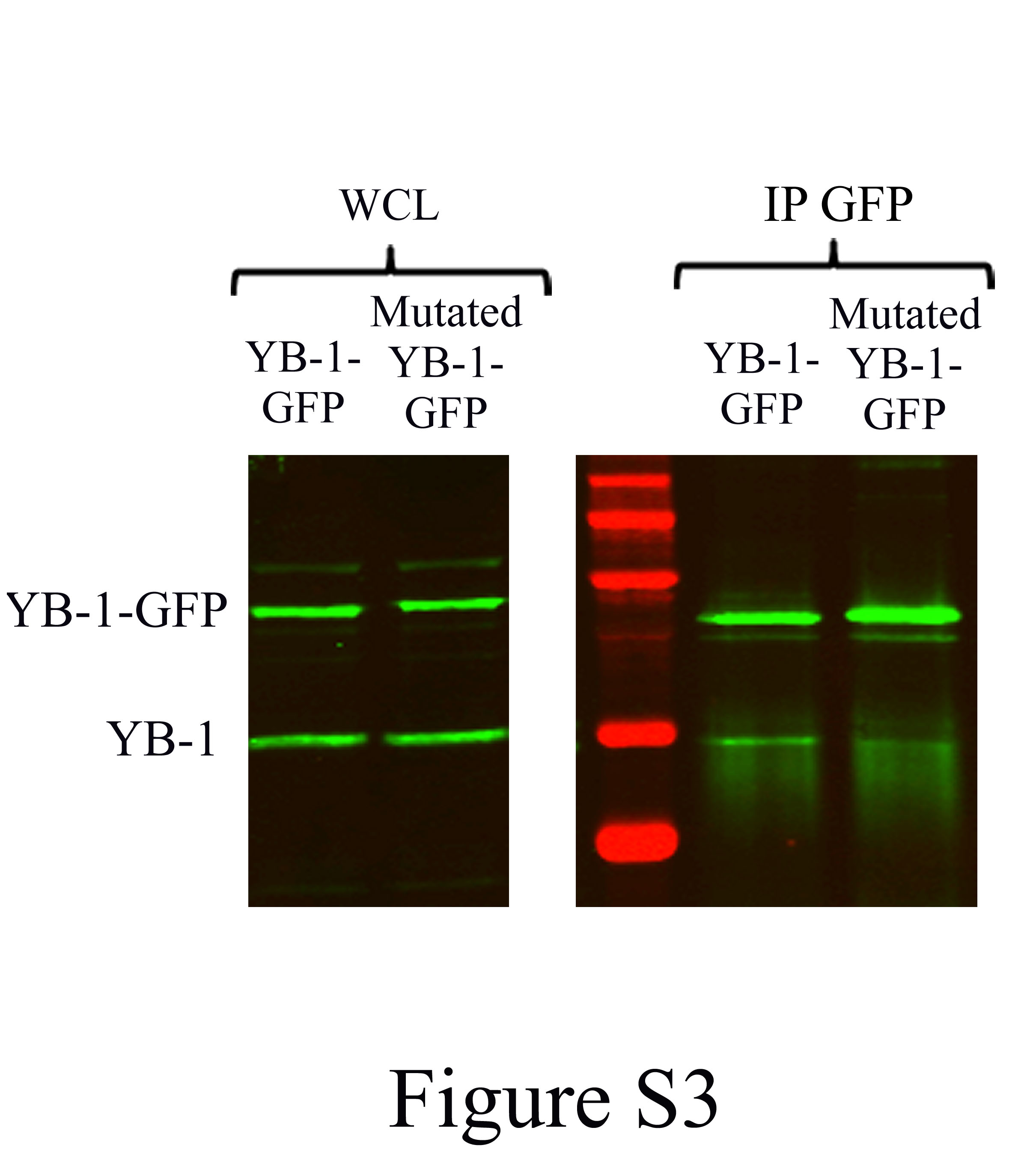

Supplement: Supplementary Figure S3 [file srep17304-s7.jpg]

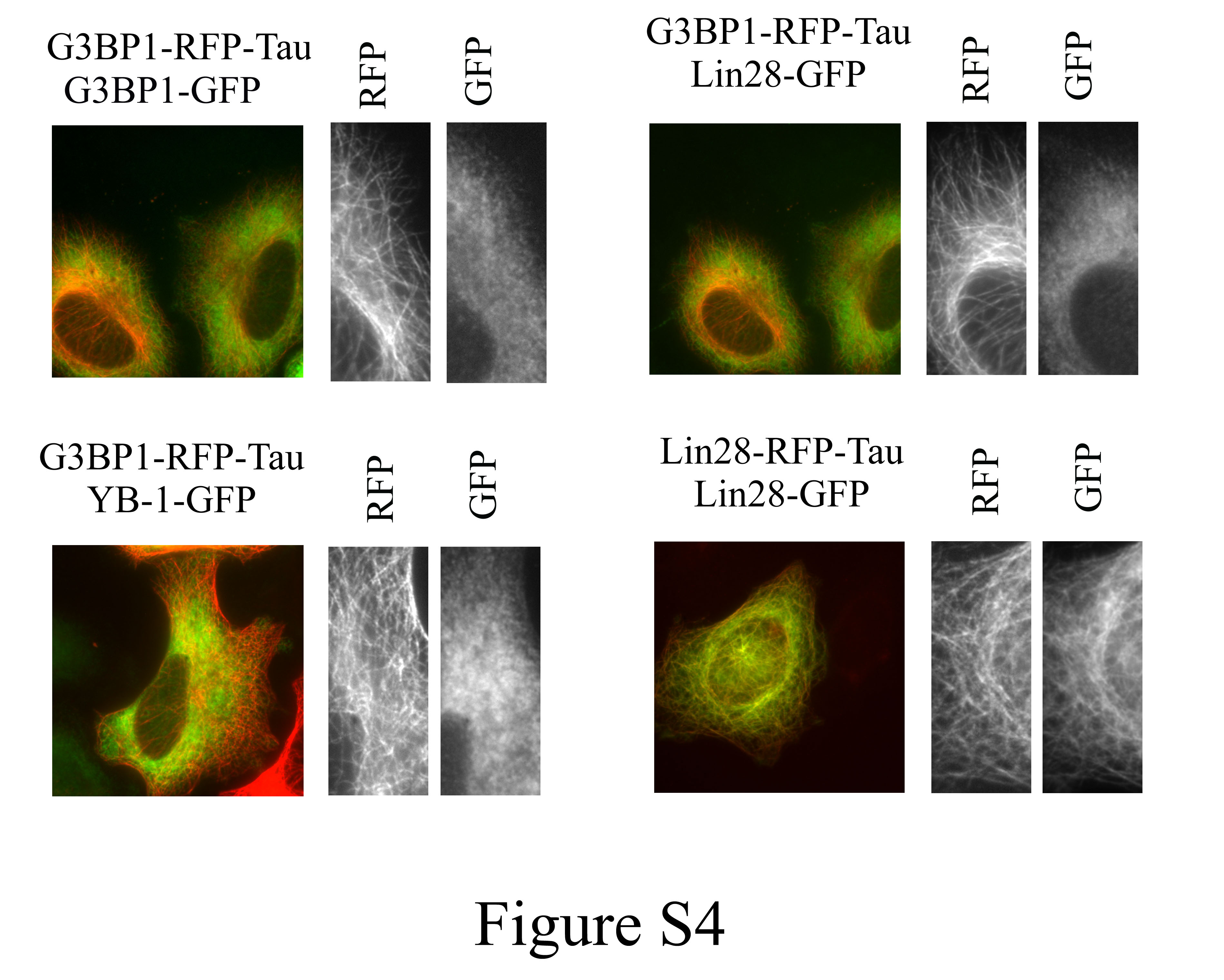

Supplement: Supplementary Figure S4 [file srep17304-s8.jpg]

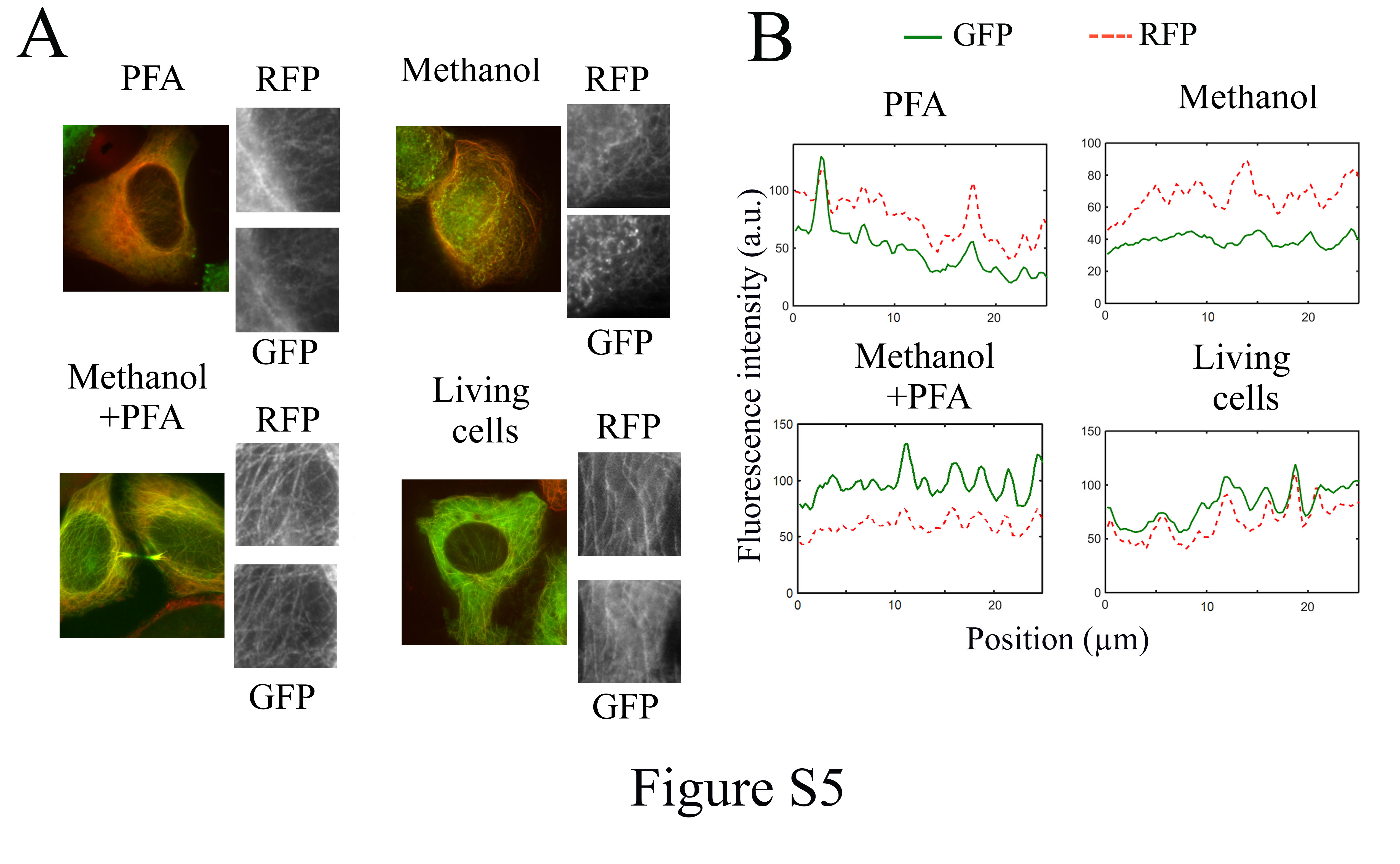

Supplement: Supplementary Figure S5 [file srep17304-s9.jpg]

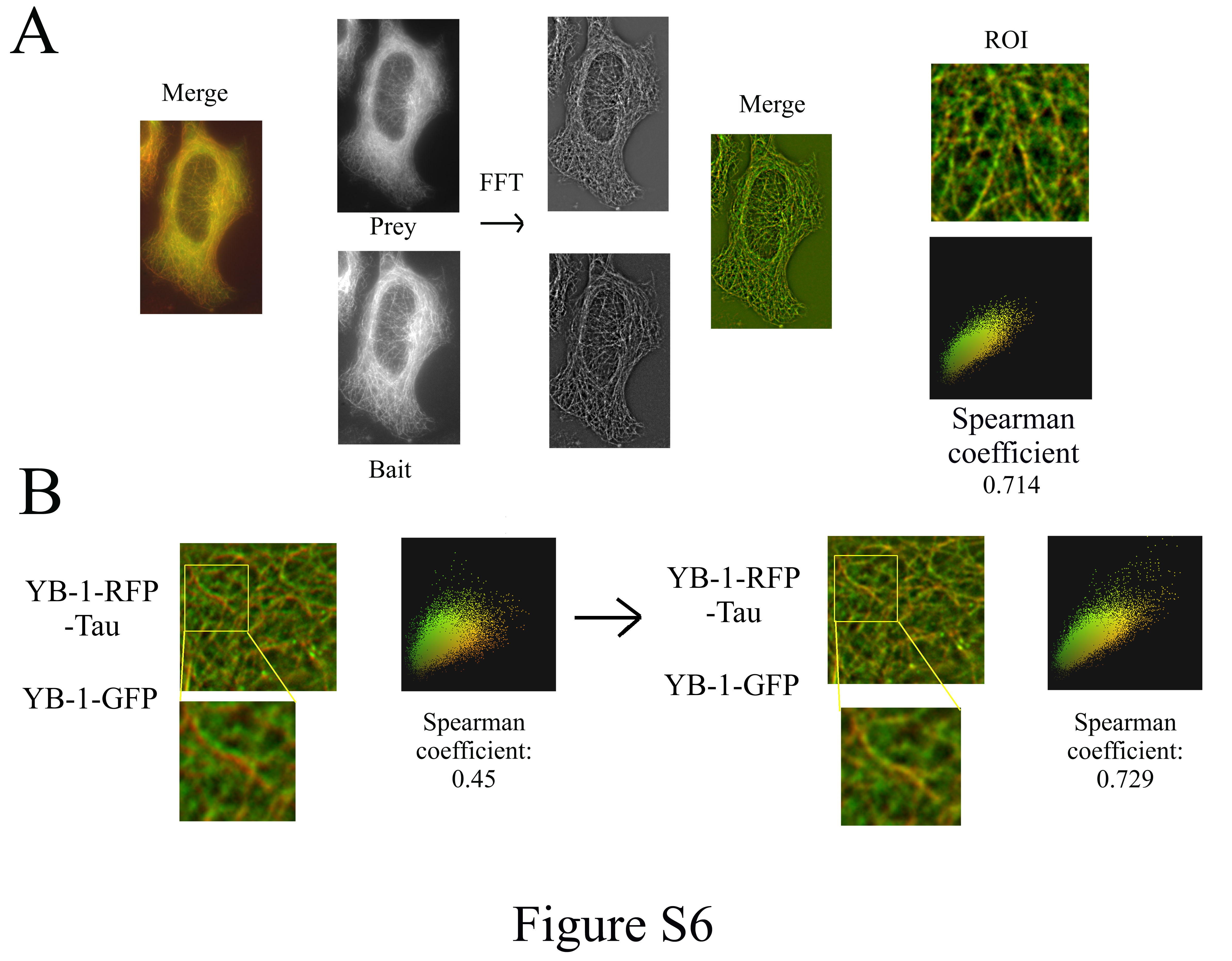

Supplement: Supplementary Figure S6 [file srep17304-s10.jpg]
